# Effects of Intracoronary Alteplase on Microvascular Function in Acute Myocardial Infarction

**DOI:** 10.1161/JAHA.119.014066

**Published:** 2020-01-28

**Authors:** Annette M. Maznyczka, Peter J. McCartney, Keith G. Oldroyd, Mitchell Lindsay, Margaret McEntegart, Hany Eteiba, Paul Rocchiccioli, Richard Good, Aadil Shaukat, Keith Robertson, Vivek Kodoth, John P. Greenwood, James M. Cotton, Stuart Hood, Stuart Watkins, Peter W. Macfarlane, Julie Kennedy, R. Campbell Tait, Paul Welsh, Naveed Sattar, Damien Collison, Lynsey Gillespie, Alex McConnachie, Colin Berry

**Affiliations:** ^1^ British Heart Foundation Glasgow Cardiovascular Research Centre Institute of Cardiovascular and Medical Sciences University of Glasgow Glasgow United Kingdom; ^2^ West of Scotland Heart and Lung Centre Golden Jubilee National Hospital, Clydebank Glasgow United Kingdom; ^3^ Leeds University and Leeds Teaching Hospitals NHS Trust Leeds United Kingdom; ^4^ Wolverhampton University Hospital NHS Trust Wolverhampton United Kingdom; ^5^ Electrocardiology Group Royal Infirmary Glasgow United Kingdom; ^6^ Department of Haematology Royal Infirmary Glasgow United Kingdom; ^7^ Project Management Unit Greater Glasgow and Clyde Health Board Glasgow United Kingdom; ^8^ Robertson Centre for Biostatistics Institute of Health and Wellbeing, University of Glasgow Glasgow United Kingdom

**Keywords:** cardiovascular magnetic resonance, fibrinolysis, microvascular obstruction, primary PCI, ST‐segment–elevation myocardial infarction, Myocardial Infarction, Percutaneous Coronary Intervention, Treatment

## Abstract

**Background:**

Impaired microcirculatory reperfusion worsens prognosis following acute ST‐segment–elevation myocardial infarction. In the T‐TIME (A Trial of Low‐Dose Adjunctive Alteplase During Primary PCI) trial, microvascular obstruction on cardiovascular magnetic resonance imaging did not differ with adjunctive, low‐dose, intracoronary alteplase (10 or 20 mg) versus placebo during primary percutaneous coronary intervention. We evaluated the effects of intracoronary alteplase, during primary percutaneous coronary intervention, on the index of microcirculatory resistance, coronary flow reserve, and resistive reserve ratio.

**Methods and Results:**

A prespecified physiology substudy of the T‐TIME trial. From 2016 to 2017, patients with ST‐segment–elevation myocardial infarction ≤6 hours from symptom onset were randomized in a double‐blind study to receive alteplase 20 mg, alteplase 10 mg, or placebo infused into the culprit artery postreperfusion, but prestenting. Index of microcirculatory resistance, coronary flow reserve, and resistive reserve ratio were measured after percutaneous coronary intervention. Cardiovascular magnetic resonance was performed at 2 to 7 days and 3 months. Analyses in relation to ischemic time (<2, 2–4, and ≥4 hours) were prespecified. One hundred forty‐four patients (mean age, 59±11 years; 80% male) were prospectively enrolled, representing 33% of the overall population (n=440). Overall, index of microcirculatory resistance (median, 29.5; interquartile range, 17.0–55.0), coronary flow reserve(1.4 [1.1–2.0]), and resistive reserve ratio (1.7 [1.3–2.3]) at the end of percutaneous coronary intervention did not differ between treatment groups. Interactions were observed between ischemic time and alteplase for coronary flow reserve (*P*=0.013), resistive reserve ratio (*P*=0.026), and microvascular obstruction (*P*=0.022), but not index of microcirculatory resistance.

**Conclusions:**

In ST‐segment–elevation myocardial infarction with ischemic time ≤6 hours, there was overall no difference in microvascular function with alteplase versus placebo.

**Clinical Trial Registration:**

URL: https://www.clinicaltrials.gov. Unique identifier: NCT02257294.

**Clinical Perspective**

**What Is New?**

- Overall, there was no difference in index of microcirculatory resistance, coronary flow reserve, or resistive reserve ratio with alteplase versus placebo.
- Patients presenting with a limited ischemic time (<2 hours) had dose‐related improvements in microvascular function (coronary flow reserve and resistive reserve ratio) with alteplase versus placebo.
- Patients presenting with longer ischemic times (≥4 hours) had increased microvascular obstruction extent with alteplase versus placebo.

**What Are the Clinical Implications?**

- The findings are relevant to ongoing trials of intracoronary fibrinolytics during primary percutaneous coronary intervention, and to clinicians considering bail‐out lytic therapy in acute ST‐segment–elevation myocardial infarction patients, with high thrombus burden and no reflow.

## Introduction

Failed myocardial reperfusion affects approximately half of patients with acute ST‐segment–elevation myocardial infarction (STEMI) following primary percutaneous coronary intervention (PCI)^1^ and microvascular obstruction confers a worse prognosis.^2^, ^3^, ^4^ Microvascular obstruction is an acute, but potentially reversible, pathology. The pathophysiology includes microvascular thrombi, endothelial disruption, and, if reperfusion does not occur, then irreversible hemorrhagic transformation occurs within the infarct core.^5^

Facilitated PCI with full‐ or half‐dose fibrinolytic therapy given intravenously pre‐PCI improves initial coronary flow, but promotes paradoxical thrombus formation, which counteracts fibrinolysis and is associated with higher residual thrombus burden, thrombotic complications, bleeding, and mortality.^6^, ^7^, ^8^ In the T‐TIME (A Trial of Low‐Dose Adjunctive Alteplase During Primary PCI) trial (NCT02257294), we hypothesized that low‐dose, intracoronary fibrinolysis with adequate anticoagulation would reduce intracoronary and microvascular thrombosis and distal embolization without activating thrombus formation, thereby reducing microvascular obstruction. However, as assessed by contrast‐enhanced cardiovascular magnetic resonance (CMR) imaging, microvascular obstruction did not differ with low‐dose intracoronary alteplase versus placebo.^9^

In contrast with CMR, the index of microcirculatory resistance (IMR) quantifies immediate efficacy of microcirculatory reperfusion.^10^, ^11^ Elevated IMR is quantitatively associated with microvascular obstruction,^12^, ^13^ myocardial hemorrhage,^12^ worse recovery of infarct size,^14^ and adverse left ventricular (LV) remodeling and function.^15^ In acute STEMI, an IMR ≤32 post‐PCI predicts recovery of LV function^16^ whereas an IMR ≥32 predicts all‐cause death or rehospitalization for heart failure.^13^ An IMR >40 after primary PCI has been associated with all‐cause death, heart failure readmissions, and major adverse cardiac events.^13^, ^14^, ^15^

Resistive reserve ratio (RRR) measures the vasodilatory capacity of the coronary microcirculation. It is the ratio between basal resting tone and resistance at maximal hyperemia and is lower when microvasodilatation is impaired. Coronary flow reserve (CFR) reflects epicardial and microcirculatory vasodilator capacity, as well as residual epicardial stenosis. CFR is the ratio of hyperemic to resting coronary blood flow. In acute STEMI, a lower RRR and CFR predict microvascular obstruction^12^, ^17^ and larger infarction.^17^, ^18^

We prospectively investigated the effects of intracoronary alteplase during primary PCI on acute invasive parameters of microcirculatory function. We hypothesized that intracoronary alteplase would be associated with lower IMR, higher CFR, and higher RRR. Second, we hypothesized that the effects of alteplase on IMR, CFR, and RRR may vary with ischemic time, TIMI (Thrombolysis in Myocardial Infarction) thrombus grade, and TIMI coronary flow grade at the time of drug delivery (prespecified interaction analyses).

## Methods

The data that support the findings of this study are available from the corresponding author upon reasonable request.

### Study Design and Patient Selection

**Figure 1:**
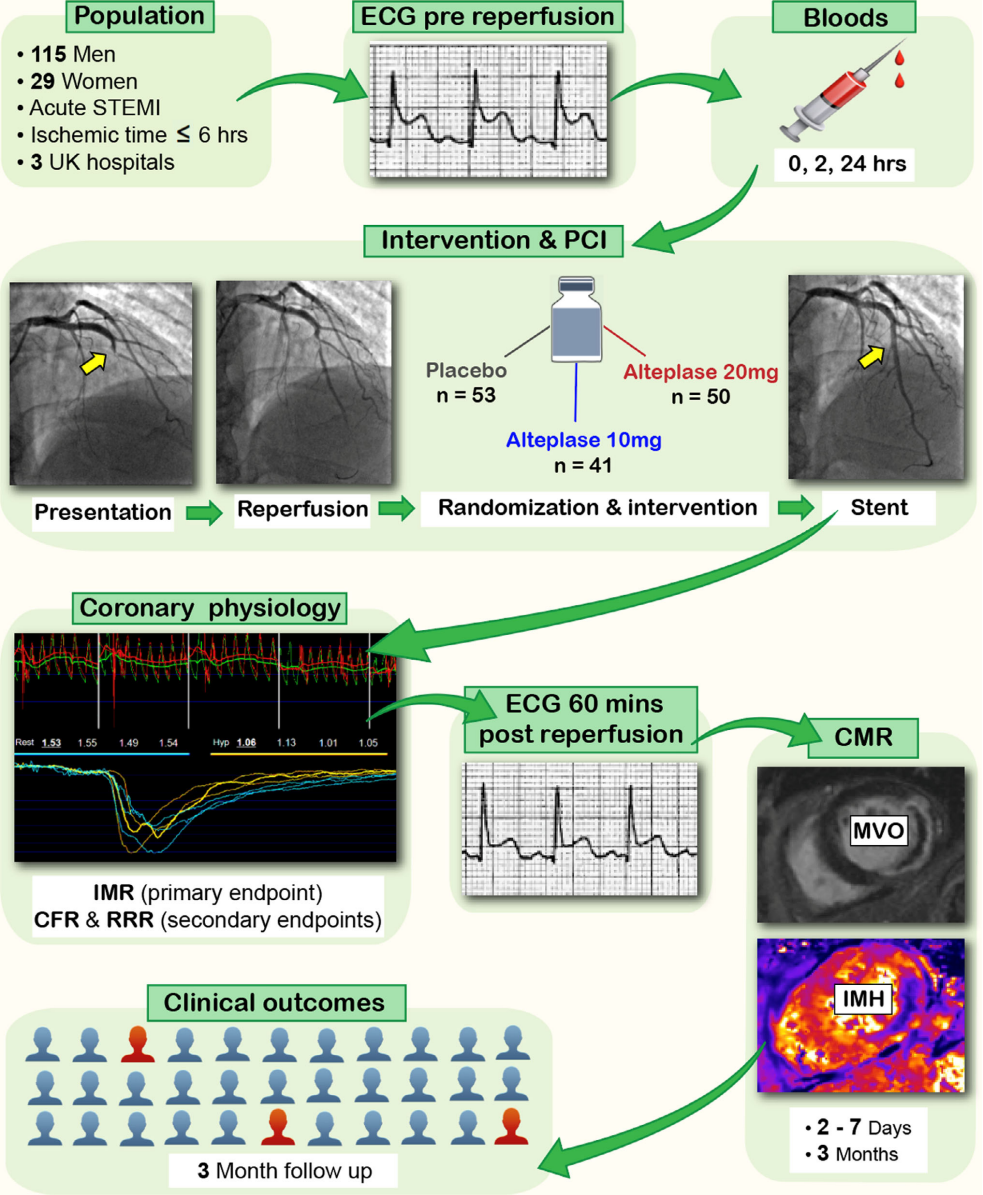
Illustration of the study design. Acute STEMI patients meeting the eligibility criteria were enrolled in the catheterization laboratory and randomized to placebo, alteplase 10 mg, or alteplase 20 mg, in a 1:1:1 dose‐ranging, double‐blind design. CFR indicates coronary flow reserve; IMH, intramyocardial hemorrhage; IMR, index of microcirculatory resistance; MVO, microvascular obstruction; PCI, percutaneous coronary intervention; RRR, resistive reserve ratio; STEMI, ST‐segment–elevation myocardial infarction.

We performed a predefined, prospective, nested substudy within the main T‐TIME trial. From August 2016 to December 2017, patients with acute STEMI from 3 hospitals in the United Kingdom were randomized in a 1:1:1 dose‐ranging, double‐blind study. The protocol is summarized in Figure 1. Participants were treated according to contemporary practice guideline recommendations.^4^

Patients were eligible for study participation if they presented with persistent ST‐segment elevation or recent left bundle branch block within 6 hours of symptom onset and either an occluded culprit artery (TIMI coronary flow grade ≤1) or reduced flow (TIMI flow grade 2, slow but complete filling) in the presence of angiographic evidence of thrombus (TIMI thrombus grade ≥2). Eligibility required radial artery access, and the occlusion had to be in the proximal or mid segment of a major coronary artery. Exclusion criteria included a functional coronary collateral supply (Rentrop grade ≥2) to the culprit artery, cardiogenic shock, comorbidities with expected survival <1 year and contraindications to fibrinolysis, or CMR. The full list of eligibility criteria is provided in Data S1.

Enrollment into the physiology study within the 3 designated sites was based on prospective assessment of eligibility criteria, operator experience, and logistical considerations at the point of care. A screening register was prospectively completed to document the reasons for participation or not. Witnessed verbal assent to participate in the study was obtained in the catheterization laboratory, and written informed consent was subsequently obtained on the ward. The study complied with the Declaration of Helsinki^19^ and was approved by the West of Scotland Research Ethics Committee (reference 13‐WS‐0119).

### Randomization and Intervention

Patients were randomized using an interactive voice‐response–based system. The randomization sequence was computer generated, using the method of randomized permuted blocks of length 6, with stratification by location of STEMI (anterior versus nonanterior). The allocation sequence was on a 1:1:1 basis between placebo and the reduced dose alteplase groups (10, 20 mg; ie, one‐tenth or one‐fifth of the standard dose). Patients, staff, and researchers were blinded to treatment group allocation.

After successful reperfusion (TIMI flow grade ≥2) was achieved, using balloon angioplasty and/or aspiration thrombectomy, patients received the allocated intervention immediately in the catheterization laboratory. The 20‐mL volume of study drug was manually infused into the culprit coronary artery over 5 to 10 minutes proximal to the culprit lesion, using either an intracoronary catheter or the guiding catheter if selectively engaged.

### Intracoronary Physiology Measurements and Analysis

IMR, CFR, and RRR were measured at the end of primary PCI, using a pressure‐ and temperature‐sensing guidewire (Abbott Vascular, Santa Clara, CA). All patients received 200 μg of intracoronary nitroglycerin in the culprit artery. The calibrated wire was equalized to guide catheter pressure, then advanced to the distal third of the culprit artery. Using standard thermodilution methodology, mean transit time (Tmn) of a hand‐injected 3‐mL bolus of room‐temperature saline was measured in triplicate at rest and during steady‐state maximal hyperemia induced by intravenous adenosine (140 μg/kg/min). In order to mitigate the possibility of bias through disclosure of the IMR, CFR, and RRR results, the operators were blinded. The blinding process involved obscuring the display of the RadiAnalyzer Xpress monitor by turning it away from the clinician and other clinical staff. Experienced physiology technicians recorded the thermodilution data and quality assured the acquisition.

IMR was quantified by distal coronary pressure × Tmn during hyperemia.^20^ At the end of primary PCI when IMR was measured after stenting, there was no residual epicardial stenosis, and therefore IMR correction with coronary wedge pressure^21^ or Yong's formula^22^ was not required. CFR was quantified by dividing resting Tmn by hyperemic Tmn.^23^, ^24^ RRR was quantified by dividing the baseline resistance index (distal coronary pressure × Tmn under resting conditions) by IMR.^25^ The shape of the hyperemic thermodilution curves was assessed as narrow unimodal, wide unimodal, or bimodal.^26^ A narrow unimodal waveform was defined as an acute temperature reduction (duration of <0.42 seconds from the beginning of the temperature reduction to nadir temperature), followed by rapid return to resting temperature. A wide unimodal waveform was defined as a temperature decrease to nadir >0.42 seconds, followed by a gradual return to baseline temperature. A bimodal waveform was defined as having 2 distinct nadirs, with a valley deeper than 20% of peak temperature drop.

All data were extracted from the RadiAnalyzer Xpress instrument and then analyzed offline using Coroflow software (Coroventis Research AB, Uppsala, Sweden) by an investigator blinded to treatment allocation and blinded to CMR data. The coronary physiology data were subject to a second read, and final data were established by consensus agreement.

### Angiographic, ECG, and Troponin Analyses

ECG and angiographic end points were determined by blinded core laboratory analysis, using standard operating procedures.

Angiograms were analyzed by A.M.M. and then subject to a second read by 1 of 2 interventional cardiologists, both with >10 years of experience. Discrepancies were resolved by consensus agreement between the first and second reviewers, or where discrepancies remained consensus was reached after discussion with a third reviewer. The following were assessed in the culprit artery: TIMI coronary flow grade, TIMI frame count, myocardial perfusion grade, and TIMI thrombus grade. The angiogram acquisition protocol required stored fluoroscopy of study drug administration to enable verification by the core laboratory that the guide catheter was selectively engaged in the culprit artery when used to deliver study drug. Angiographic methods are described in detail in Data S1.

The absolute percentage ST‐segment resolution on ECGs obtained 60 minutes after reperfusion compared with prereperfusion was calculated. Troponin T area under the curve was measured from blood samples obtained immediately prereperfusion (0 hours) and then again 2  and 24 hours later.

### Cardiovascular Magnetic Resonance

CMR imaging was performed at 1.5 Tesla. The standard operating procedure for CMR included: (1) microvascular obstruction presence and extent (% LV mass) demonstrated by late gadolinium enhancement images; (2) myocardial hemorrhage presence and extent (% LV mass) demonstrated by T_2_* mapped images; (3) infarct size (% LV mass) demonstrated by late gadolinium enhancement images; and (4) LV ejection fraction. Microvascular obstruction and myocardial hemorrhage were reported 2 to 7 days post‐STEMI; the other CMR parameters were reported at 2 to 7 days and 3 months post‐STEMI. A detailed description of the CMR acquisition and analysis techniques are in Data S1.

### Coagulation

Coagulation and hemostasis parameters were measured in peripheral blood samples taken prereperfusion, then 2 and 24 hours postreperfusion. The parameters included fibrinogen and plasminogen (measures of systemic fibrinolysis), fibrin D‐dimer (a measure of fibrin lysis), tissue plasminogen activator (a measure of endogenous fibrinolytic system activation and circulating alteplase), and prothrombin fragment F_1+2_ (a measure of thrombin activation).

### Clinical Outcomes

Clinical outcomes were prospectively collected between the index event and 3‐month follow‐up. Major adverse cardiac events was defined as cardiovascular death, nonfatal myocardial infarction, or unplanned hospitalization for heart failure. All‐cause death and heart failure hospitalization were also reported. All events were adjudicated by a clinical event committee who were independent of the trial and blinded to the treatment allocation.

### Sample Size

Sample‐size calculation was based on data from the MR‐MI (Magnetic Resonance Imaging in Acute ST‐Segment Elevation Myocardial Infarction) cohort study^12^, ^15^ in patients who fulfilled the eligibility criteria for T‐TIME. For a comparison of IMR between 3 groups (placebo versus alteplase 10 mg versus alteplase 20 mg), assuming a mean IMR of 33.9 and an SD of 25.2, and assuming mean differences in IMR between the 10 and 20 mg alteplase groups versus placebo of 10 and 20, respectively, then 108 subjects (36/group) were needed for 85% power and a significance level of 0.05. For a comparison of CFR between 3 groups (placebo versus alteplase 10 mg versus alteplase 20 mg), assuming a mean CFR of 1.65 and an SD of 0.8, and assuming mean differences in CFR between the 10 and 20 mg alteplase groups versus placebo were 0.4 and 0.8, respectively, then 69 subjects (n=23/group) were needed with 85% power (α=0.05).

### Statistical Analysis

Analyses were performed according to treatment received (alteplase 10 mg, 20 mg, or placebo). Primary and secondary outcomes were assessed using linear regression (continuous outcomes), logistic regression (binary outcomes), or proportional odds logistic regression (ordinal outcomes) to make treatment effect estimates. In linear regression models, logarithmic or square root transformations were used, where necessary, to improve model residual distributions. We performed post hoc analyses in prespecified subgroups. We prespecified subgroups of interest according to patient characteristics: (1) ischemic time (<2, 2–4, and ≥4 hours); (2) TIMI thrombus grade immediately prestudy drug (≤2 and ≥3); and (3) TIMI coronary flow grade immediately prestudy drug (≤2, and 3). These subgroups were based on an a priori concern that they were clinically relevant patient characteristics that could potentially impact on associations of alteplase with IMR, CFR, and RRR. Regression models were used to assess treatment effects within prespecified subgroups through use of treatment‐by‐subgroup interactions. Interaction test *P* values, reported from regression models, included treatment group as a 3‐level categorical variable or as a 2‐level categorical variable (active versus placebo) and treatment modeled as a linear trend across dose groups (0, 10, and 20 mg). Regression analyses were adjusted for location of the myocardial infarction. All tests were 2‐tailed and assessed at the 5% significance level. There was no imputation for missing values, and no adjustments for multiple statistical comparisons were made. Data were analyzed using R software (R Development Core Team, Los Angeles, CA), according to a statistical analysis plan that was finalized before data lock.

## Results

### Study Population Characteristics

**Figure 2:**
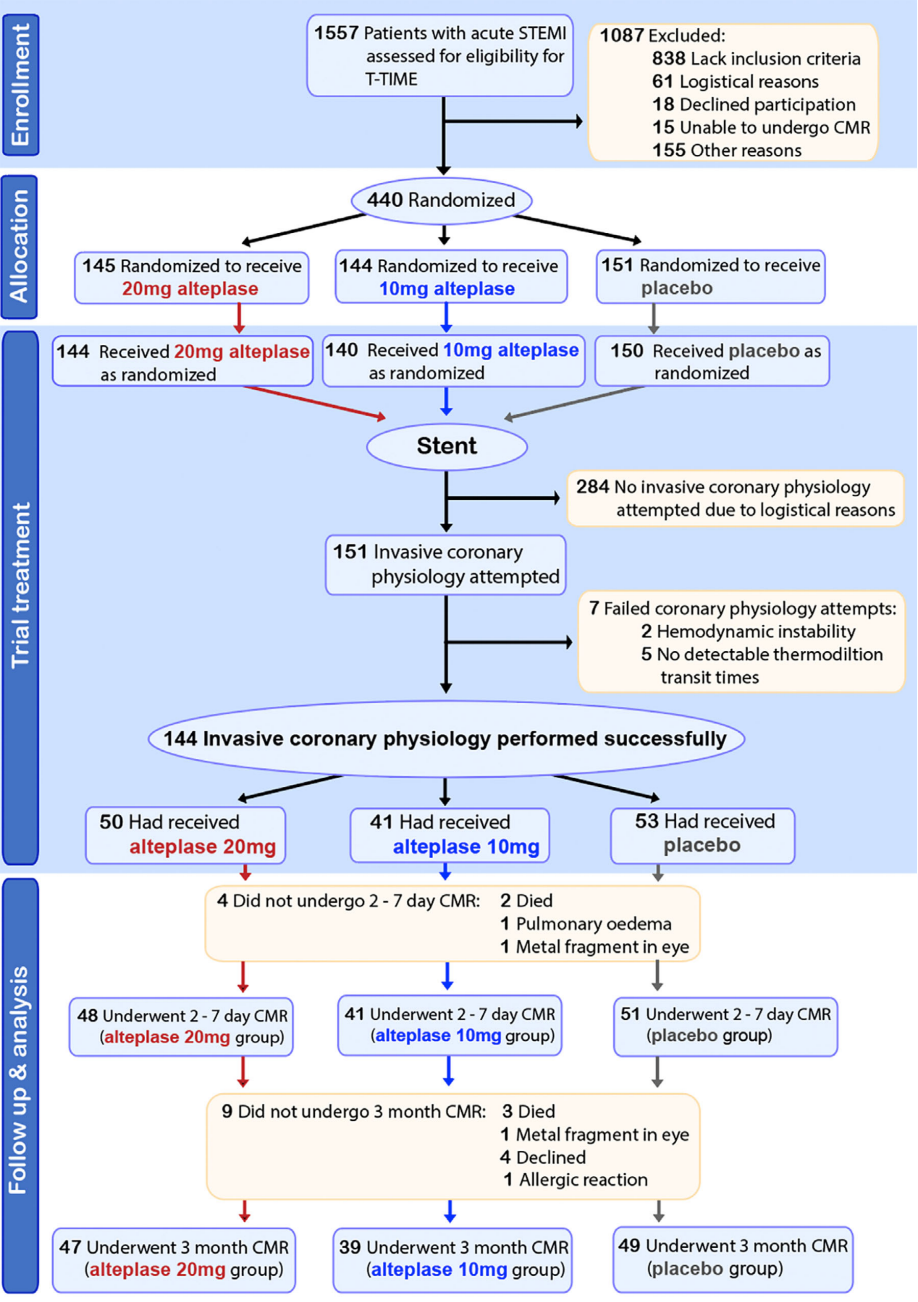
Diagram showing patient recruitment, randomization, and flow through the T‐TIME physiology substudy. CMR indicates cardiovascular magnetic resonance; STEMI, ST‐segment–elevation myocardial infarction; T‐TIME, A Trial of Low‐Dose Adjunctive Alteplase During Primary PCI.

Participants’ characteristics are shown in Tables 1 and 2. The flow of subjects through the study is summarized in Figure 2. The sample size (n=144) represented 33% of the overall study population, and their characteristics were broadly similar. Mean age was 59.4±10.5 years, and 80% were male. Median ischemic time was 2.5 hours (interquartile range [IQR], 2.0, 3.5), and 31 (22%) had an ischemic time <2 hours. The culprit coronary artery was the left anterior descending in 38% (n=54), circumflex in 17% (n=24), and right in 46% (n=66) of patients.

**Table 1:** Population Characteristics

	All [n=144]	Placebo [n=53]	Alteplase 10 mg [n=41]	Alteplase 20 mg [n=50]
Demographics				
Age, y	59.4±10.5	56.8±11.3	61.2±9.4	60.6±10.3
Male	115 (80%)	45 (85%)	31 (76%)	39 (78%)
BMI, kg/m^2^	28.4±5.1	28.8±5.3	29.0±5.2	27.4±4.6
Ischemic time, h:mm median (IQR)	2:47 (2:03, 3:50)	2:40 (2:03, 3:52)	2:43 (1:53, 4:10)	2:54 (2:10, 3:36)
Medical history				
Hypertension	41 (28%)	14 (26%)	11 (27%)	16 (32%)
Hypercholesterolemia	21 (15%)	11 (21%)	6 (15%)	4 (8%)
Diabetes mellitus^a^	16 (11%)	6 (11%)	6 (15%)	4 (8%)
Smoking				
Current	68 (47%)	25 (47%)	17 (41%)	26 (52%)
Former	27 (19%)	13 (25%)	7 (17%)	7 (14%)
Never	49 (34%)	15 (28%)	17 (41%)	17 (34%)
Previous PCI	9 (6%)	3 (6%)	1 (2%)	5 (10%)
Previous MI	8 (6%)	2 (4%)	1 (2%)	5 (10%)
Angina	4 (3%)	2 (4%)	0 (0%)	2 (4%)
Stroke/TIA	3 (2%)	2 (4%)	0 (0%)	1 (2%)
Pre‐existing maintenance medication				
Aspirin	20 (14%)	8 (15%)	4 (10%)	8 (16%)
P2Y_12_ inhibitor				
Clopidogrel	1 (1%)	1 (2%)	0 (0%)	0 (0%)
Ticagrelor/prasugrel	2 (1%)	0 (0%)	1 (2%)	1 (2%)
Statin	25 (17%)	13 (25%)	6 (15%)	6 (12%)
Βeta‐blocker	14 (10%)	4 (8%)	4 (10%)	6 (12%)
ACEi or ARB	18 (12%)	7 (13%)	4 (10%)	7 (14%)
MRA	3 (2%)	1 (2%)	2 (5%)	0 (0%)
Hemodynamic measures and initial blood results on admission				
Heart rate, bpm	73.0±15.1	74.4±16.1	71.5±13.0	72.7±15.8
Systolic BP, mm Hg	140±26	140±28	141±23	138±27
Diastolic BP, mm Hg	82±15	84±17	82±13	81±16
Creatinine, μmol/L^b^	79±16	79±16	80±14	79±17
eGFR, mL/min/1.73 m^2^ b	91±21	94±22	88±18	92±22
Hemoglobin, g/L^b^	145.8±13.7	145.4±13.8	146.2±12.6	146.0±14.6
Platelet count, 10^9^/L^b^	264.2±62.1	252.4±53.6	280.9±73.5	262.9±58.1

Data are mean±SD, or n (%), unless otherwise stated. ACEi indicates angiotensin‐converting enzyme inhibitor; ARB, angiotensin receptor blocker; BMI, body mass index; BP, blood pressure; eGFR, estimated glomerular filtration rate; IQR, interquartile range; MI, myocardial infarction; MRA, mineralocorticoid receptor antagonist; PCI, percutaneous coronary intervention; TIA, transient ischemic attack.

a Diabetes mellitus was defined as a history of diet‐controlled or treated diabetes mellitus.

b Missing data: creatinine, eGFR, hemoglobin, and platelets, 1 subject (alteplase 20 mg group).

**Table 2:** Procedure Characteristics

	All [n=144]	Placebo [n=53]	Alteplase 10 mg [n=41]	Alteplase 20 mg [n=50]
Culprit artery				
LAD	54 (38%)	19 (36%)	17 (41%)	18 (36%)
Circumflex	24 (17%)	9 (17%)	9 (22%)	6 (12%)
RCA	66 (46%)	25 (47%)	15 (37%)	26 (52%)
Culprit artery diameter, mm	3.2 ±0.4	3.2±0.5	3.2±0.5	3.2±0.4
Balloon angioplasty prestent	141 (98%)	51 (96%)	41 (100%)	49 (98%)
Initial angiography				
TIMI coronary flow grade				
0	114 (79%)	47 (89%)	34 (83%)	33 (66%)
1	14 (10%)	2 (4%)	3 (7%)	9 (18%)
2	16 (11%)	4 (8%)	4 (10%)	8 (16%)
3	0 (0%)	0 (0%)	0 (0%)	0 (0%)
TIMI thrombus grade				
3	3 (2%)	0 (0%)	0 (0%)	3 (6%)
4	25 (17%)	6 (11%)	5 (12%)	14 (28%)
5	116 (81%)	47 (89%)	36 (88%)	33 (66%)
Immediately prestudy drug				
TIMI coronary flow grade^a^				
1	5 (4%)	2 (4%)	2 (5%)	1 (2%)
2	41 (29%)	14 (27%)	10 (24%)	17 (35%)
3	95 (67%)	35 (69%)	29 (71%)	31 (63%)
TIMI thrombus grade^a^				
1	12 (9%)	5 (10%)	2 (5%)	5 (10%)
2	21 (15%)	10 (20%)	7 (17%)	4 (8%)
3	62 (44%)	21 (41%)	21 (51%)	20 (41%)
4	46 (33%)	15 (29%)	11 (27%)	20 (41%)
Study drug administration				
Thrombectomy catheter	106 (74%)	39 (74%)	29 (71%)	38 (76%)
Guide catheter	35 (24%)	13 (25%)	11 (27%)	11 (22%)
Other	3 (2%)	1 (2%)	1 (2%)	1 (2%)
Poststudy drug				
PCI with stent implant	144 (100%)	53 (100%)	41 (100%)	50 (100%)
Total stent length, mm	35.6±13.2	33.6±12.2	38.1±14.6	35.8±13.0
Poststent dilatation	133 (92%)	46 (87%)	41 (100%)	46 (92%)
Acute therapy following first medical contact^b^				
Aspirin loading dose				
300 mg	142 (99%)	53 (100%)	40 (98%)	49 (98%)
None	2 (1%)	0 (0%)	1 (2%)	1 (2%)
Additional antiplatelet medication				
None	2 (1%)	0 (0%)	1 (2%)	1 (2%)
Clopidogrel	87 (60%)	27 (51%)	28 (68%)	32 (64%)
Ticagrelor	55 (38%)	26 (49%)	12 (29%)	17 (34%)
Prasugrel	0 (0%)	0 (0%)	0 (0%)	0 (0%)
Unfractionated heparin, U median (IQR)	11 500 (10 000, 15 000)	10 000 (10 000, 14 000)	12 000 (10 000, 15 000)	12 000 (10 000, 15 375)
Intravenous morphine	134 (93%)	48 (91%)	38 (93%)	48 (96%)
Inhaled oxygen	20 (14%)	8 (15%)	7 (17%)	5 (10%)
Glycoprotein IIb/IIIa antagonist	8 (6%)	1 (2%)	5 (12%)	2 (4%)
Aspiration thrombectomy	23 (16%)	8 (15%)	8 (20%)	7 (14%)

Data are mean±SD, or n (%), unless otherwise stated. IQR indicates interquartile range; LAD, left anterior descending artery; PCI, percutaneous coronary intervention; RCA, right coronary artery; TIMI, Thrombolysis in Myocardial Infarction.

a Missing data: TIMI coronary flow grade and TIMI thrombus grade immediately prestudy drug, 3 subjects (2 placebo, 1 alteplase 20 mg group).

b None of the patients received intravenous or intracoronary treatment with bivalirudin, metoprolol, nicorandil, or sodium nitroprusside.

At initial coronary angiography, TIMI flow grade was ≤1 in 124 (88%) patients and 2 in 20 (12%) patients. The thrombus grade at presentation was 5 (occluded vessel) in 116 (81%) patients, 4 (thrombus dimension >2 vessel diameters) in 25 (17%) patients, and 3 (thrombus dimension >1/2 and <2 vessel diameters) in 3 (2%) patients. The residual thrombus grade postreperfusion immediately before study drug delivery was 4 in 46 (33%) patients, 3 in 62 (44%) patients, 2 in 21 (15%) patients, and 1 in 12 (9%) patients. All but one of the patients received the study intervention according to the protocol.

### Primary Physiology End Point

Median IMR for the entire population was 29.5 (IQR, 17.0, 55.0). Forty‐eight percent (n=69) had an IMR >32 at the end of the procedure, and 40% (n=57) had an IMR >40. On logistic regression analysis, older age was the only baseline characteristic that independently predicted an IMR >32 (mean age was 57.2±9.5 in those with IMR ≤32 and mean age was 61.7±11.2 in those with IMR >32). Overall, IMR did not differ between the alteplase groups (10, 20 mg) and placebo (alteplase 10 mg: median 22.0 [IQR, 17.0, 42.0] versus placebo, 33.0 [17.0, 57.0]; relative difference, 0.79 [95% CI, 0.58, 1.07]; *P*=0.125; alteplase 20 mg: 37.0 [20.0–57.8] versus placebo, 33.0; relative difference, 1.04 [95% CI, 0.78–1.38]; *P*=0.801; Table 3).

**Table 3:** Coronary Physiology End Points

	Treatment Group				Treatment Effect			
	All (n=144)	Placebo (n=53)	Alteplase 10 mg (n=41)	Alteplase 20 mg (n=50)	20 mg vs Placebo	10 mg vs Placebo	10 or 20 mg vs Placebo	Trend With Dose
					Estimate (95% CI) *P* Value	Estimate (95% CI) *P* Value	Estimate (95% CI) *P* Value	Estimate (95% CI) *P* Value
IMR^a^	29.5 (17.0, 55.0)	33.0 (17.0, 57.0)	22.0 (17.0, 42.0)	37.0 (20.0, 57.8)	1.04 (0.78, 1.38) *P*=0.801	0.79 (0.58, 1.07) *P*=0.125	0.92 (0.71, 1.18) *P*=0.505	1.02 (0.88, 1.17) *P*=0.824
IMR >40^b^	57 (40%)	24 (45%)	11 (27%)	22 (44%)	0.93 (0.42, 2.05) *P*=0.864	0.42 (0.17, 1.02) *P*=0.054	0.66 (0.33, 1.34) *P*=0.251	0.96 (0.64, 1.43) *P*=0.840
IMR >32^b^	69 (48%)	27 (51%)	15 (37%)	27 (54%)	1.12 (0.51, 2.44) *P*=0.774	0.54 (0.23, 1.24) *P*=0.147	0.81 (0.41, 1.60) *P*=0.546	1.05 (0.71, 1.55) *P*=0.794
CFR^a^	1.4 (1.1, 2.0)	1.3 (1.1, 1.8)	1.4 (1.1, 1.9)	1.4 (1.1, 2.0)	1.03 (0.88, 1.20) *P*=0.732	1.01 (0.86, 1.19) *P*=0.900	1.02 (0.89, 1.17) *P*=0.777	1.01 (0.94, 1.09) *P*=0.732
CFR ≤2^b^	115 (80%)	44 (83%)	31 (76%)	40 (80%)	1.23 (0.45, 3.36) *P*=0.680	1.62 (0.59, 3.36) *P*=0.680	1.40 (0.58, 3.36) *P*=0.451	1.11 (0.68, 1.79) *P*=0.681
RRR^a^	1.6 (1.3, 2.3)	1.6 (1.3, 2.2)	1.6 (1.4, 2.6)	1.8 (1.3, 2.4)	1.02 (0.87, 1.20) *P*=0.795	1.04 (0.88, 1.23) p=0.658	1.03 (0.90, 1.18) =0.685	1.01 (0.93, 1.09) 0.790
Waveform^c^								
Unimodal (narrow)	75 (52%)	26 (49%)	22 (54%)	27 (54%)	0.98 (0.46, 2.06) *P*=0.956	1.00 (0.45, 2.20) *P*=0.999	0.99 (0.52, 1.89) *P*=0.972	0.99 (0.68, 1.44) *P*=0.957
Unimodal (wide)	56 (39%)	25 (47%)	14 (34%)	17 (34%)				
Bimodal	13 (9%)	2 (4%)	5 (12%)	6 (12%)				
LVEDP, mm Hg^d^	17.0 (12.0, 20.8)	16.5 (13.2, 19.0)	19.0 (13.2, 22.8)	15.0 (12.0, 18.8)	0.89 (0.75, 1.05) *P*=0.172	1.05 (0.88, 1.25) *P*=0.579	0.96 (0.83, 1.12) *P*=0.609	0.94 (0.87, 1.03) *P*=0.184

Data are median (IQR) or n (%). Between‐group comparisons derived from linear, logistic, or ordinal logistic regression models, adjusted for location of MI (see footnotes). CFR indicates coronary flow reserve; IMR, index of microcirculatory resistance; IQR, interquartile range; LVEDP, left ventricular end‐diastolic pressure; MI, myocardial infarction; RRR, resistive reserve ratio.

a Data analyzed on logarithmic scale. Treatment effect estimates reported as relative differences between groups, with 95% CI and *P* value, from linear regression model adjusted for location of MI.

b Treatment effect estimates reported as odds ratios between groups, with 95% CI and *P* value, from logistic regression model adjusted for location of MI.

c Treatment effect estimates reported as odds ratio between groups, with 95% CI and *P* value, from ordinal logistic regression model adjusted for location of MI.

d Missing data: LVEDP, 18 subjects (7 placebo, 3 alteplase 10 mg, and 8 alteplase 20 mg group).

The intraclass correlation coefficient (ICC) for IMR, assessed from 30 consecutive patients, showed excellent intrarater reliability (ICC, 0.998 [95% CI, 0.997, 0.999]) and inter‐rater reliability (ICC, 0.999 [95% CI, 0.998, 0.999]). The mean difference between repeated IMR measurements from 12 patients was 6.33 (*P*=0.076).

### Secondary Physiology End Points

Median CFR for the population was 1.4 (IQR, 1.1, 2.0). At the end of the procedure, 115 (88%) patients had a CFR ≤2.0. The median RRR for the entire population was 1.7 (IQR, 1.3, 2.3). Overall, neither CFR nor RRR differed with alteplase versus placebo (Table 3). The mean difference between repeated CFR measurements from 9 patients was 0.07 (*P*=0.659). The mean difference between repeated RRR measurements from 9 patients was −0.04 (*P*=0.860). The ICC for RRR, assessed from 30 consecutive patients, showed excellent intrarater reliability (ICC, 0.988 [95% CI, 0.974, 0.994]) and inter‐rater reliability (ICC, 0.988 [95% CI, 0.975, 0.994]).

Thermodilution waveforms or LV end‐diastolic pressure did not differ with alteplase versus placebo.

### Angiographic, ECG, and Troponin Results

As in the main trial, there were no differences in final TIMI coronary flow grade or TIMI frame count between treatment groups (Table 4). TIMI myocardial perfusion grade was higher in the alteplase 20 mg group compared with the placebo group (odds ratio, 2.16 [95% CI, 1.04, 4.49]; *P*=0.039). There was no difference in TIMI myocardial perfusion grade between the alteplase 10 mg versus placebo group (odds ratio, 1.32 [95% CI, 0.60, 2.92]; *P*=0.496). Percent ST‐segment resolution 60 minutes postreperfusion did not differ with alteplase versus placebo (Table 4). Troponin T under the curve (0–24 hours) did not differ with alteplase versus placebo (Table 4).

**Table 4:** ECG, Angiographic, and Troponin End Points

	Treatment Group				Treatment Effect			
	All (n=144)	Placebo (n=53)	Alteplase	Alteplase	20 mg vs Placebo	10 mg vs Placebo	10 or 20 mg vs Placebo	Trend With Dose
			10 mg (n=41)	20 mg (n=50)	Estimate (95% CI) *P* Value	Estimate (95% CI) *P* Value	Estimate (95% CI) *P* Value	Estimate (95% CI) *P* Value
Absolute % ST‐segment resolution 60 min^a^^,^ b	46.6 (40.9)	45.1 (37.8)	45.7 (43.8)	48.8 (42.4)	4.15 (−11.71, 20.02) *P*=0.608	1.32 (−15.53, 18.16) *P*=0.878	2.89 (−11.04, 16.83) *P*=0.684	2.08(−5.83, 9.98) *P*=0.607
TIMI flow grade post‐PCI^c^					1.70 (0.51, 5.69) *P*=0.391	1.43 (0.42, 4.84) *P*=0.565	1.57 (0.57, 4.32) *P*=0.387	1.31 (0.71, 2.41) *P*=0.383
1	3 (2%)	2 (4%)	0	1 (2%)				
2	15 (10%)	6 (11%)	5 (12%)	4 (8%)				
3	126 (88%)	45 (85%)	36 (88%)	45 (90%)				
TIMI MPG post‐PCI^c^								
0	42 (29)	18 (34)	15 (37)	9 (18)	2.16 (1.04, 4.49) *P*=0.039 §	1.32 (0.60, 2.92) *P*=0.496	1.75 (0.91, 3.37) *P*=0.091	1.47 (1.02, 2.21) *P*=0.039 §
1	3 (2)	3 (6)	0	0				
2	60 (42)	19 (36)	15 (37)	26 (52)				
3	39 (27)	13 (25)	11 (27)	15 (30)				
TFC post‐PCI, median (IQR)^d^	18.0 (14.0, 26.0)	18.0 (14.0, 26.0)	16.5 (14.0, 22.4)	22.0 (14.0, 24.5)	1.03 (0.84, 1.27) *P*=0.774	0.89 (0.72, 1.11) *P*=0.311	0.97 (0.80, 1.16) *P*=0.713	1.01 (0.91, 1.13) *P*=0.789
Troponin T AUC 0 to 24 h (mg/L)^c^^,^ d	125.6 (143.2)	115.6 (139.5)	130.8 (142.4)	131.7 (150.5)	1.39 (0.83, 2.34) *P*=0.213	1.56 (0.91, 2.67) *P*=0.110	1.46 (0.93, 2.30) *P*=0.098	1.18 (0.91, 1.53) *P*=0.206

Data are mean±SD, or n (%), unless otherwise stated. Between‐group comparisons derived from linear, logistic, or ordinal logistic regression models, adjusted for location of MI (see footnotes). AUC indicates area under the curve; IQR, interquartile range; MI, myocardial infarction; MPG, myocardial perfusion grade; PCI, percutaneous coronary intervention; TFC, TIMI frame count; TIMI, Thrombolysis in Myocardial Infarction.

a Treatment effect estimates reported as mean differences between groups, with 95% CI and *P* value, from linear regression model adjusted for MI location.

b Missing data: ST‐segment resolution 60 min, 3 subjects (2 placebo, 1 alteplase 10 mg group). Troponin T AUC, 21 subjects (8 placebo, 5 alteplase 10 mg, and 8 alteplase 20 mg group).

c Treatment effect estimates reported as odds ratio between groups, with 95% CI and *P* value, from a proportional odds logistic regression model, adjusting for MI location.

d Data analyzed on a logarithmic scale. Treatment effect estimates reported as relative difference between groups, with 95% CI and *P* value, from linear regression model adjusted for MI location.

### Cardiovascular Magnetic Resonance Imaging Results

CMR was performed in 140 patients (97%) from 2 to 7 days after enrollment and in 135 patients (94%) at 3 months. Overall, there was no difference in microvascular obstruction or myocardial hemorrhage presence or extent, infarct size, myocardial salvage, LV ejection fraction, or volumes with alteplase versus placebo (Table S1).

### Coagulation and Hematological Variables

There was an alteplase dose‐related increase in systemic concentrations of fibrin D‐dimer, reflecting fibrinoylsis, and prothrombin fragment F_1+2_, reflecting activation of the clotting system, and a reduction in plasminogen, reflecting the intended effect of alteplase (Table S2). Systemic concentrations of fibrinogen and hemoglobin were similar between treatment groups (Table S2), indicating that effects of alteplase were localized to the heart.

### Clinical Outcomes

Follow‐up information was available for all patients at 3 months post‐STEMI. Major adverse cardiac events occurred in 20 patients by 3 months, of whom 7 received placebo, 7 received alteplase 10 mg, and 6 received alteplase 20 mg. Three patients died during the 3‐month follow‐up period, of whom 1 received alteplase 10 mg and 2 received alteplase 20 mg. There were 17 unplanned hospitalizations for heart failure by 3 months (7 in the placebo group, 6 in the alteplase 10 mg group, and 4 in the alteplase 20 mg group).

### Subgroup Analyses

**Figure 3:**
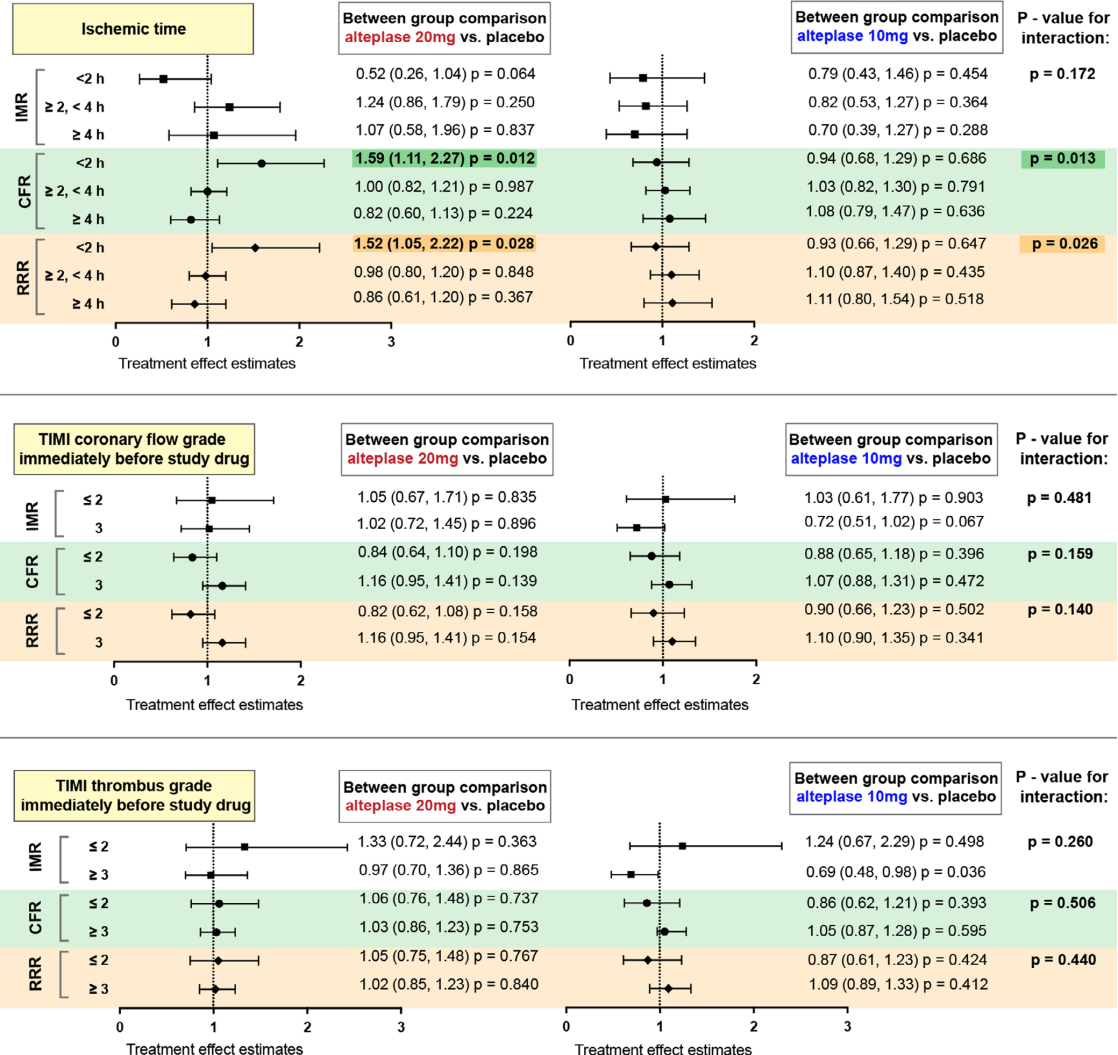
Forest plots showing treatment effect estimates and interaction *P* values for IMR (index of microcirculatory resistance), CFR (coronary flow reserve), and RRR (resistive reserve ratio) in subgroups for ischemic time and TIMI (Thrombolysis in Myocardial Infarction) coronary flow grade and thrombus grade immediately before study drug delivery.

Treatment effect estimates for IMR, CFR, and RRR measured in the culprit coronary artery at the end of PCI in the prespecified subgroups, based on postulated pathophysiological mechanisms, are shown in Figure 3, Table 5, and Tables S3 through S4.

**Table 5:** IMR, CFR, and RRR, Microvascular Obstruction and Myocardial Hemorrhage in Subgroups of Ischemic Time

Ischemic Time	Placebo [n=53]	Alteplase 10 mg [n=41]	Alteplase 20 mg [n=50]	Treatment Effect		Interaction *P* Value (treatment as 3‐level categorical variable)	Treatment Effect	Interaction *P* Value (treatment as 2‐level categorical variable)	Treatment Effect	Interaction *P* Value (treatment as per 10 mg increase in dose)
				20 mg vs Placebo	10 mg vs Placebo		10 or 20 mg vs Placebo		Trend With Dose	
IMR										
<2 h [n]	45.0 (23.0, 53.2) [10]	29.0 (17.0, 36.0) [13]	19.5 (15.0, 22.8) [8]	0.52 (0.26, 1.04) *P*=0.064	0.79 (0.43, 1.46) *P*=0.454	0.172	0.68 (0.39, 1.19) *P*=0.173	0.367	0.73 (0.52, 1.02) *P*=0.068	0.097
≥2, <4 h [n]	31.5 (15.5, 51.0) [30]	24.0 (15.0, 42.0) [17]	39.5 (20.0, 61.2) [32]	1.24 (0.86, 1.79) *P*=0.250	0.82 (0.53, 1.27) *P*=0.364		1.07 (0.76, 1.51) *P*=0.685		1.12 (0.93, 1.34) *P*=0.243	
≥4 h [n]	28.0 (19.0, 60.0) [13]	19.0 (17.0, 34.0) [11]	51.0 (26.8, 59.0) [10]	1.07 (0.58, 1.96) *P*=0.837	0.70 (0.39, 1.27) *P*=0.238		0.86 (0.51, 1.44) *P*=0.556		1.02 (0.75, 1.38) *P*=0.918	
CFR										
<2 h [n]	1.2 (1.1, 1.7) [10]	1.4 (1.0, 1.8) [13]	2.0 (1.8, 2.3) [8]	1.59 (1.11, 2.27) *P*=0.012*	0.94 (0.68, 1.29) *P*=0.686	0.013*	1.14(0.85, 1.55) *P*=0.379	0.652	1.24 (1.04, 1.49) *P*=0.019*	0.038*
≥2, <4 h [n]	1.3 (1.1, 1.8) [30]	1.3 (1.2, 1.8) [17]	1.4 (1.2, 2.0) [32]	1.00 (0.82, 1.21) *P*=0.987	1.03 (0.82, 1.30) *P*=0.791		1.01 (0.84, 1.21) *P* =0.915		1.00 (0.91, 1.10) 0.984	
≥4 h [n]	1.7 (1.4, 2.0) [13]	1.8 (1.3, 2.6) [11]	1.2 (1.0, 1.6) [10]	0.82 (0.60, 1.13) *P*=0.224	1.08 (0.79, 1.47) *P*=0.636		0.95 (0.72, 1.25) *P*=0.697		0.91 (0.78, 1.07) *P*=0.264	
RRR										
<2 h [n]	1.5 (1.3, 1.9) [10]	1.6 (1.1, 2.2) [13]	2.2 (2.0, 2.6) [8]	1.52 (1.05, 2.22) *P*=0.028*	0.93 (0.66, 1.29) *P*=0.647	0.026*	1.12 (0.82, 1.53) *P*=0.481	0.827	1.22 (1.01, 1.47) *P*=0.041*	0.093
≥2, <4 h [n]	1.6 (1.3, 2.2) [30]	1.6 (1.5, 2.6) [17]	1.6 (1.3, 2.2) [32]	0.98 (0.80, 1.20) *P*=0.848	1.10 (0.87, 1.40) *P*=0.435		1.02 (0.85, 1.23) *P*=0.833		0.99 (0.89, 1.1.0) *P*=0.838	
≥4 h [n]	1.9 (1.6, 2.3) [13]	2.3 (1.6, 2.8) [11]	1.4 (1.0, 2.5) [10]	0.86 (0.61, 1.20) *P*=0.367	1.11 (0.80, 1.54) *P*=0.518		0.98 (0.74, 1.31) *P*=0.907		0.93 (0.79, 1.1.0) *P*=0.420	
<2 hours [n]	2.1±3.9 [9]	2.2±4.0 [13]	0.0±0.0 [8]	−0.87 (−2.07, 0.33) *P*=0.155	−0.03 (−1.10, 1.03) *P*=0.950	0.039*	−0.35 (−1.34, 0.64) *P*=0.484	0.089	−0.42(−1.02, 0.17) *P*=0.161	0.010*
≥2, <4 h [n]	2.4±3.3 [29]	2.6±4.4 [17]	2.8±5.9 [31]	−0.14 (−0.77, 0.50) *P*=0.676	−0.14 (−0.90, 0.61) *P*=0.715		−0.14 (−0.72, 0.45) *P*=0.643		−0.07 (−0.38, 0.25) *P*=0.676	
≥4 h [n]	0.9±1.6 [13]	2.8±4.5 [11]	6.0 ±6.6 [9]	1.47 (0.40, 0.54) *P*=0.007*	0.49 (−0.52, 1.50) *P*=0.337		0.93 (0.05, 1.82) 0.039*		0.72 (0.19, 1.25) *P*=0.007*	
<2 h [n]	0.5±0.9 [7]	1.7±3.8 [11]	0.0±0.0 [8]	−0.51 (−4.44, 3.42) *P*=0.800	1.20 (−2.48, 4.87) *P*=0.523	0.153	0.48 (−2.91, 3.86) *P* =0.783	0.392	−0.30 (−2.25, 1.66) *P*=0.766	0.080
≥2, <4 h [n]	2.2±3.3 [28]	2.7±4.4 [15]	2.2±4.9 [31]	−0.06 (−2.05, 1.92) *P*=0.950	0.41 (−2.04, 2.86) *P*=0.741		0.09 (−1.75, 1.93) *P*=0.926		−0.04 (−1.02, 0.95) *P*=0.941	
≥4 h [n]	0.8±1.7 [12]	1.6±3.1 [10]	5.1±5.9 [9]	4.25 (0.88, 7.61) *P*=0.013*	0.80 (−2.46, 4.06) *P*=0.632		2.42 (−0.41, 5.25) *P*=0.094		2.06 (0.39, 3.73) *P*=0.016	

Data are median (IQR) or mean±SD. Coronary physiology data were analyzed on a logarithmic scale, with treatment effect estimates reported as relative differences and 95% CIs with *P* values, derived from linear regression, adjusted for location of MI. Microvascular obstruction (% LV) was analyzed on a square root scale, with treatment effect estimates reported as mean differences and 95% CIs with *P* values, derived from linear regression, adjusted for location of MI. CFR indicates coronary flow reserve; IMR, index of microcirculatory resistance; IQR, interquartile range; LV, left ventricular; MI, myocardial infarction; RRR, resistive reserve ratio.

#### Ischemic time

There was no interaction between ischemic time and alteplase with IMR (Table 5). In patients with an ischemic time <2 hours, median IMR was 45.0 (23.0, 53.2) with placebo, 29.0 (17.0, 36.0) with alteplase 10 mg, and 19.5 (15.0, 22.8) with alteplase 20 mg. In patients with an ischemic time ≥4 hours, median IMR was 28.0 (19.0, 60.0) with placebo, 19.0 (17.0, 34.0) with alteplase 10 mg, and 51.0 (26.8, 59.0) with alteplase 20 mg.

Interactions were observed between ischemic time and alteplase for CFR (*P*=0.013) and RRR (*P*=0.026; Figure 3; Table 5). In patients with ischemic times <2 hours, median CFR was higher with alteplase (placebo, 1.2 [1.1, 1.7]; alteplase 10 mg, 1.4 [1.0, 1.8]; alteplase 20 mg, 2.0 [1.8, 2.3]). RRR was also higher with alteplase in patients with an ischemic time <2 hours (placebo, 1.5 [1.3, 1.9]; alteplase 10 mg, 1.6 [1.1, 2.2]; alteplase 20 mg, 2.2 [2.0, 2.6]). In patients with an ischemic time ≥4 hours, RRR was 2.0 (1.4, 2.7) with placebo, 2.3 (1.6, 2.8) with alteplase 10 mg, and 1.4 (1.0, 2.5) with alteplase 20 mg. In those with an ischemic time ≥4 hours, CFR was 1.7 (1.4, 2.0) with placebo, 1.8 (1.3, 2.6) with alteplase 10 mg, and 1.2 (1.0, 1.6) with alteplase 20 mg.

An interaction occurred between ischemic time and alteplase for amount of microvascular obstruction (*P*=0.022). In patients with an ischemic time ≥4 hours, alteplase increased the mean extent of microvascular obstruction: placebo (0.89 ± 1.65%), alteplase 10 mg (2.77 ± 4.54%), and alteplase 20 mg (5.97 ± 6.58%). In those with an ischemic time <2 hours, mean amount of microvascular obstruction was: 2.06 ± 3.93% with placebo, 2.22 ± 3.98% with alteplase 10 mg, and 0.00 ± 0.00% with alteplase 20 mg.

#### TIMI flow and thrombus grades prestudy drug

There was no interaction between treatment group and IMR, CFR, or RRR in the following subgroups: (1) TIMI coronary flow grade immediately prestudy drug dichotomized by ≤2 or 3; (2) TIMI thrombus grade immediately prestudy drug dichotomized by ≤2 or ≥3 (Figure 3 and Tables S3 and S4).

## Discussion

The main finding is that overall microvascular function, assessed by IMR, CFR, and RRR, did not differ between alteplase and placebo groups.

The lack of an overall treatment effect on microvascular function in the culprit artery contrasts with the findings of Sezer et al.^27^ In their proof‐of‐concept study, low‐dose intracoronary thrombolysis (streptokinase, 250 kU; n=51) was infused over 3 minutes through a guide catheter at the end of primary PCI and, when compared with standard care (n=44), resulted in a significant increase in CFR and decrease in IMR (CFR, 2.5 versus 1.7; *P*<0.001; IMR, 20.2 versus 34.2; *P*<0.001).^27^

There are important differences between our study and that of Sezer et al.^27^ First, the study by Sezer et al^27^ was not double blinded, whereas our study was. Second, streptokinase is not fibrin specific, whereas alteplase is. Third, all of the patients in Sezer et al's study^27^ received a bolus of tirofiban glycoprotein IIbIIIa inhibitor therapy at the start of the procedure followed by tirofiban infusion for 12 hours, whereas only 6% of patients in our study received a glycoprotein IIbIIIa inhibitor, in line with current practice guideline recommendations.^28^ Fourth, streptokinase was delivered poststent when 89% of the cohort had TIMI 3 coronary flow, whereas we administered alteplase prestent. In our study, 46 patients (32%) had TIMI coronary flow ≤2 immediately prestudy drug, which may have limited alteplase reaching the downstream microcirculation, and prothrombotic effects of fibrinolytics might be enhanced in conditions of slow flow.^29^, ^30^ Fifth, we measured coronary physiology immediately after the primary PCI procedure, whereas Sezer et al^27^ measured IMR and CFR 48 hours after primary PCI, when IMR and CFR may have undergone partial recovery.^31^, ^32^, ^33^

In exploratory prespecified subgroup analyses, which were intended to provide mechanistic insights and should be interpreted as hypothesis generating, interactions were observed between ischemic time and alteplase with CFR and RRR, but not IMR. The improvement in microvascular vasodilator function with alteplase, as reflected by higher CFR and RRR, in patients with ischemic time <2 hours may be explained by those patients presenting with a brief ischemic time having intact microcirculation, which was modifiable by therapy, whereas those with a longer ischemic time may have had irreversible microvascular injury. While these observations could be attributed to type 1 statistical error, our findings are supported by consistent effects of ischemic time on extent of microvascular obstruction, for which the *P* value for interaction was significant.

In our study, alteplase was associated with more microvascular obstruction in patients with an ischemic time ≥4 hours. The findings suggest the possibility of alteplase having a detrimental effect on myocardial reperfusion in patients with longer ischemic time. The mechanism may involve alteplase promoting myocardial hemorrhage in circumstances of prolonged ischemia, characterized by capillary degradation^34^ and myocyte necrosis. An increase in extravasation of blood into the interstitial space of the infarct core results in external compression of capillaries, with an associated increase in microvascular resistance. This leads to more microvascular obstruction and potentiates the progression of myocardial hemorrhage. The findings support the rationale to limit eligibility to a short ischemic time (eg, <4 hours).

A plausible explanation for the lack of interaction between ischemic time and alteplase with IMR could be because IMR measures microvascular resistance during maximal hyperemia, which might be less modifiable by intracoronary alteplase than microvascular vasodilator function (measured by RRR and CFR).

Two other ongoing trials of low‐dose intracoronary fibrinolysis are using IMR as an eligibility criterion and to measure acute microvascular function after intervention. The RESTORE‐MI (Restoring Microcirculatory Perfusion in STEMI) trial (ACTRN12618000778280) will randomize STEMI patients with IMR >32 (n=800) to intracoronary tenecteplase (one‐third of weight‐based systemic dose) or placebo, in a double‐blind design, and those with IMR ≤32 will continue in a followup registry. Recently, a pilot trial in 36 patients with acute STEMI with symptoms ≤ 6 hours and TIMI 0/1 flow in the culprit artery demonstrated adjunctive low‐dose (4 mg) of intracoronary tenecteplase given twice (post‐reperfusion and at the end of PCI, 8 mg total) compared with placebo (saline) as an adjunct to primary PCI was feasible and safe but did not improve percent stenosis of the culprit lesion (primary outcome).^35^ The smaller OPTIMAL (Optimal Coronary Flow After PCI for Myocardial Infarction) trial (NCT02894138) will randomize 80 STEMI patients with a poststenting IMR >30 to intracoronary alteplase (20mg), or placebo, in an open‐label design. Both studies are including patients with ischemic times up to 12 hours. However, our findings suggest that therapeutic benefit with alteplase might be restricted to patients with a shorter ischemic time.

### Limitations and Strengths

Because of the potential for type 1 error in the subgroup analyses, these should be interpreted as exploratory. Although we observed a higher TIMI myocardial perfusion grade post‐PCI in the alteplase 20 mg group compared with the placebo group, this difference was not observed in the main trial,^9^ and the significant *P* value may have occurred because of chance (type 1 statistical error).

Strengths of our study include the randomized, double‐blind design, blinding of IMR, CFR, and RRR measurements to minimize bias, and their excellent inter‐ and intrarater reliability. Retention with CMR was high (94% at 3 months).

## Conclusions

In acute STEMI with ischemic time ≤6 hours, there was, overall, no difference in culprit artery microvascular function (IMR, CFR, or RRR) at the end of PCI with alteplase versus placebo. Interactions were observed between ischemic time and alteplase on CFR, RRR, and microvascular obstruction, implying therapeutic benefit in patients presenting with a shorter ischemic time and a detrimental effect in patients with a longer ischemic time. Further research seems warranted.

## Sources of Funding

Dr Maznyczka is funded by a fellowship from the British Heart Foundation (FS/16/74/32573). Dr Berry is supported by grant RE/18/6/34217 from the British Heart Foundation. T‐TIME was supported by grant 12/170/4 from the Efficacy and Mechanism Evaluation (EME) program of the National Institute for Health Research (NIHR‐EME). Boehringer Ingelheim UK Ltd provided the study drugs (alteplase 10, 20 mg), matched placebo, and sterile water for injection. These organizations had no other involvement in the conduct of the study or in any aspect of the manuscript.

## Disclosures

Dr Berry is employed by the University of Glasgow, which holds research and/or consultancy agreements with AstraZeneca, Abbott Vascular, Boehringer Ingelheim, GSK, HeartFlow, Opsens, and Novartis. Dr Oldroyd has received speaker fees and research support from Abbott Vascular and Boston Scientific. Dr Tait reported receiving grants from the National Institute for Health Research (NIHR) during the conduct of the study, consultancy fees and honoraria from Boehringer Ingelheim and Sobi, consultancy fees and nonfinancial support from Bayer Healthcare, NovoNordisk, and Shire, consultancy fees from Pfizer, and nonfinancial support from CSL Behring. Dr Cotton reported research support and speaker fees from Abbott Vascular. Dr Watkins reports speaker fees from Biosensors International, GE Healthcare, Abbott, Sanofi, and AstraZeneca. Welsh reported receiving grants from the Chief Scientist Office, Boehringer Ingelheim, and Roche. Sattar reported receiving grants and personal fees from Boehringer Ingelheim and personal fees from Amgen, Eli Lilly, Janssen, and AstraZeneca outside the submitted work. Dr Robertson reported receiving speaker fees and educational support from AstraZeneca and educational support from Abbott Vascular.

## Supporting information


**Data S1.** Eligibility Criteria.
**Table S1.** CMR End Points
**Table S2.** Coagulation and Hematological Variables
**Table S3.** IMR, CFR, and RRR in Subgroups of TIMI Coronary Flow Grade Immediately Before Study Drug Delivery
**Table S4.** IMR, CFR, and RRR in Subgroups of TIMI Thrombus Grade Immediately Before Study Drug Delivery
