# Supplementary material for: Effects of Intracoronary Alteplase on Microvascular Function in Acute Myocardial Infarction
Source: J Am Heart Assoc. 2020 Jan 28;9(3):e014066. doi: 10.1161/JAHA.119.014066 (PMC7033872; doi:10.1161/JAHA.119.014066)
Supplement: Supplementary file 1 — Data S1. Eligibility Criteria. Table S1. CMR End Points Table S2. Coagulation and Hematological Variables Table S3. IMR, CFR, and RRR in Subgroups of TIMI Coronary Flow Grade Immediately Before Study Drug Delivery Table S4. IMR, CFR, and RRR in Subgroups of TIMI Thrombus Grade Immediately Before Study Drug Delivery [file JAH3-9-e014066-s001.pdf]

SUPPLEMENTAL MATERIAL

Data S1.

Eligibility Criteria

Patients with a clinical diagnosis of acute ST-segment elevation myocardial infarction (STEMI) were eligible for randomization according to the following eligibility criteria:

Inclusion

- Acute MI (symptom onset ≤ 6 hours) with persistent ST-segment elevation or recent left bundle branch block
- Coronary artery occlusion (TIMI [Thrombolysis in Myocardial Infarction] coronary flow grade 0 or 1), or impaired coronary flow (TIMI coronary flow grade 2, slow but complete filling) in the presence of definite angiographic evidence of thrombus (TIMI grade 2 or more)
- Proximal-mid culprit lesion location in a major coronary artery (i.e. the right, left anterior descending, intermediate, or circumflex artery)
- Radial artery access
- Successful coronary reperfusion (TIMI coronary flow grade ≥2) pre-stent achieved prior to randomization.
- Informed consent, i.e. only patients who were sufficiently well to understand the information about the study, as described by the attending cardiologist, were eligible to participate.

Exclusion

- Normal flow in the culprit coronary artery at initial angiography (TIMI grade 3)
- Functional coronary collateral supply (Rentrop grade 2/3) to the culprit artery
- Previous infarction in the culprit artery (known or suspected clinically, e.g. wall motion abnormality revealed by echocardiography)
- Cardiogenic shock (Killip Class IV)

- Multivessel percutaneous coronary intervention (PCI) intended before the day 2-7 cardiovascular magnetic resonance (CMR) scan
- Estimated body weight <60 kg
- Non-cardiac co-morbidity with expected survival <1 year
- Contra-indication to contrast-enhance CMR imaging
- Pacemaker, or implantable defibrillator
- Known impaired renal function (estimated glomerular filtration rate <30ml/min)
- Significant bleeding disorder either at present or within the past 6 months
- Known hemorrhagic diathesis
- Patient with current concomitant oral anticoagulation therapy (international normalized ratio >1.3), including apixaban, dabigatran and rivaroxaban
- Any history of central nervous system damage (i.e. neoplasm, aneurysm, intracranial or spinal surgery)
- Severe hypertension (blood pressure >180/110 mmHg) not controlled by medical therapy
- Major surgery, biopsy of a parenchymal organ, or significant trauma within the past 3 months (this includes any trauma associated with the current acute MI)
- Recent trauma to the head (<2 months)
- Prolonged cardiopulmonary resuscitation (>2 minutes) within the past 2 weeks
- Acute pericarditis and/ or subacute bacterial endocarditis
- Acute pancreatitis
- Severe hepatic dysfunction, including hepatic failure, cirrhosis, portal hypertension (esophageal varices) and active hepatitis
- Active peptic ulceration
- Arterial aneurysm and known arterial/ venous malformation
- Neoplasm with increased bleeding risk

- Any known history of hemorrhagic stroke, or stroke of unknown origin
- Known history of ischemic stroke, or transient ischemic attack in the preceding 6 months
- Dementia
- Hypersensitivity to gentamicin, or natural rubber
- Incapacity, or inability to provide informed consent
- Previous randomization to this study, or participation in a study with an investigational drug, or medical device within 90 days prior to randomization
- Women of child bearing potential (i.e. pre-menopausal), or breast feeding
- Requirement for immunosuppressive therapy at any time during the preceding 3 months. This would include corticosteroids (but not inhaled or topical), drugs used following transplantation (e.g tacrolimus, cyclosporine), anti-metabolite therapies (e.g. mycophenolic acid, azathioprine, leflunomide and immunomodulators including biologics (e.g. adalimumab, or etanercept) and disease modifying anti-rheumatic drugs. This list is not exhaustive.
- Active or prophylactic treatment with oral, or parenteral antibiotic, antifungal, or antiviral therapy, to prevent or treat infection
- Any anti-cancer treatment (excluding surgery as this is covered above) at any time during the preceding 3 months, including chemotherapy, radiotherapy, and treatment with biologics, such as Vascular Endothelial Growth Factor Receptor (VEGFR) inhibitors (e.g. bevacizumab, pazopanib). This list is not exhaustive.
- Any significant concurrent, or recent condition(s) not listed above that in the opinion of the treating clinician would pose an additional risk to the patient.

Angiogram Acquisition & Analysis Methods

Coronary angiograms were acquired during emergency care with cardiac catheter laboratory X-ray and information technology equipment. The angiograms were analyzed using post-processing software (QAngio® XA Medis, Leiden, NL.) by experienced investigators who were blinded to

treatment allocation. Catheter calibration was performed using the catheter calibration function on MEDIS QAngio. For each lesion, a view perpendicular to the long axis of the vessel was used in order to avoid foreshortening and overlap of branches. The single plane projection showing the best opacified and most severe lesion with minimal foreshortening and minimal branch overlap was selected. Feedback was provided to sites on the quality and completeness of the angiograms.

TIMI Coronary Flow Grade

The TIMI coronary flow grade was assessed using the following definitions[1]:

TIMI coronary flow grade	Definition
0	No flow
1	Minimal flow past obstruction
2	Slow (but complete) filling and slow clearance
3	Normal flow and clearance

TIMI Myocardial Perfusion Grade

TIMI myocardial perfusion grade provides a score for ground-glass appearance ('blush') of the contrast entering the microvasculature and contrast washout. TIMI myocardial perfusion grade was assessed according to the following definitions[2]:

TIMI myocardial perfusion grade	Definition
0	Minimal or no myocardial blush in the distribution of the culprit artery.
1	Myocardial blush is present in the distribution of the culprit artery. But there is incomplete

	clearance of dye between injections (with ~ 30 seconds between injections).
2	Myocardial blush is present in the distribution of the culprit artery. But there is slow contrast entry into the microvasculature and slow clearance of contrast. Specifically, blush is strongly persistent (i.e. either does not or only minimally diminishes in intensity) beyond 3 cardiac cycles after injection.
3	Myocardial blush is present in the distribution of the culprit artery, with normal entry and exit of dye (mild/ moderate persistence of dye beyond 3 cardiac cycles, but notably reduced after 3 cardiac cycles). Blush that is only mild intensity throughout 3 cardiac cycles after injection (washout phase), but fades minimally is also classified as grade 3**.**

TIMI Frame Count

The TIMI frame count represents the amount of time (in frames) for contrast dye to reach a standardized distal landmark[2]. If the culprit vessel was the left anterior descending artery the frame count was divided by 1.7 (correcting for longer vessel length).

TIMI Coronary Thrombus Grade

Thrombus burden revealed during coronary angiography was classified according to the TIMI thrombus grade[3]:

Thrombus grade	Definition
0	No angiographic characteristics of thrombus are present
1	Possible thrombus is present, with reduced contrast density, haziness, irregular lesion contour, or a smooth convex ‘meniscus’ at the site of total occlusion suggestive but not diagnostic of thrombus

2	Definite thrombus, with greatest dimensions ≤ half the vessel diameter
3	Definite thrombus but with greatest long axis dimension >1/2 but <2 vessel diameters
4	Definite thrombus, with the largest dimension ≥2 vessel diameters
5	Total occlusion

CMR Acquisition & Analysis

CMR was performed using 1.5-T platforms (Siemens MAGNETOM Avanto, Erlangen, Germany and Philips Intera, Best, The Netherlands). The imaging protocol followed a standard operating procedure that included planning and localisers, T1-mapping, T2*-mapping, cine CMR with steady-state free precession (SSFP), and late gadolinium enhancement imaging 10 – 15 minutes after administration of contrast media[4]. The scan acquisitions were spatially co-registered and also included different slice orientations to enhance diagnostic confidence.

The intravenous contrast agent used in this study was gadobutrol (Gadovist®, Bayer: 1.5 mmol/ml solution for injection), which was administered in two doses. The first dose injection (0.05 mmol/kg) was given to initiate the first-pass of contrast. The second dose (0.1 mmol/kg) was given immediately after the first-pass. Therefore, the total dose of gadobutrol was 0.15 mmol/kg.

SSFP cine breath-hold sequences (with parallel imaging acceleration) were used. The heart was imaged in multiple parallel SAX planes 8-mm thick, separated by 2mm gaps, equating to approximately 10 slices and 30 cardiac phases. The CMR analyses were undertaken using Medis® Suite MR (Medis, Leiden, NL), by two trained investigators who

were blinded to treatment allocation. P.McC undertook the primary analysis of the scans and related analyses were reviewed by C.B. (second reviewer).

Late Enhancement

Late microvascular obstruction (MVO) was imaged 10-15 minutes after intravenous Gadovist contrast administration, using in general a motion corrected T1-weighted phase-sensitive inversion recovery radiofrequency pulse sequence. A full stack, aligned to T2* scans (or cines) and 3 long axis views (vertical long axis, horizontal long axis and 3 chamber view) were acquired.

MVO was defined as a dark zone on early gadolinium enhancement imaging 1, 3, 5 and 7-minutes post-contrast injection that remained present within an area of late gadolinium enhancement at 15 minutes. The endocardial and epicardial borders were contoured. The myocardial mass (grams) of the dark zone was quantified by manual delineation and expressed as a percentage of total left ventricular (LV) mass.

Infarct Definition & Size

The presence of acute infarction was established based on abnormalities in cine wall motion, rest first-pass myocardial perfusion, and late gadolinium enhancement imaging in two imaging planes. The myocardial mass of late gadolinium (grams) was quantified using computer assisted planimetry and the territory of infarction was delineated using a 5 standard deviation method and expressed as a percentage of total LV mass. Typical late gadolinium enhancement and MVO imaging parameters with phase sensitive inversion recovery: matrix 192 x 256 pixels; flip angle 25°; TE 3.36 ms; bandwidth 130 Hz/pixel; echo spacing 8.7ms and trigger pulse 2. The voxel size is 1.8 x 1.3 x 8 mm. Inversion times individually adjusted to optimize nulling of apparently normal myocardium (typical values, 200 to 300ms).

Myocardial Edema

The presence of myocardial oedema was established based on an area of increased signal intensity on the SSFP cine images (acquired two minutes after gadolinium contrast injection). The myocardial mass was calculated by manual delineation in end-diastole and end-systole. The values were averaged and expressed as a percentage of LV mass[4].

Myocardial Salvage

Myocardial salvage was calculated by subtraction of percent infarct size from percent area-at risk, as reflected by the extent of oedema. The myocardial salvage index was calculated by dividing the myocardial salvage area by the initial area-at-risk.

Myocardial Hemorrhage

On the T2* parametric maps, a threshold of 20ms was applied. A region of reduced signal intensity within the infarcted area, with a T2* value of <20 ms[5,6] was considered to confirm the presence of myocardial hemorrhage. The area was manually delineated and expressed as % LV mass.

Table S1. CMR endpoints. Data are mean ± SD, or n (%), unless otherwise stated. Between-group comparisons derived from linear, logistic, or ordinal logistic regression models, adjusted for location of MI (see footnotes).

	Treatment Group				Treatment Effect			
	All [n=144]	Placebo [n=53]	Alteplase 10mg [n=41]	Alteplase 20mg [n=50]	20mg vs. placebo Estimate (95% CI) p-value	10mg vs. placebo Estimate (95% CI) p-value	10 or 20mg vs. placebo Estimate (95% CI) p-value	Trend with dose Estimate (95% CI) p-value
**CMR 2 – 7 day**								
Microvascular obstruction extent (% LV) *† ‡	2.5 ± 4.5	2.0 ± 3.1	2.5 ± 4.2	2.9 ± 5.7	0.08 (-0.43, 0.59) p=0.766	0.03 (-0.50, 0.56) p=0.908	0.06 (-0.50, 0.50) p=0.804	0.04 (-0.21, 0.29) p=0.766
Microvascular obstruction presence * ‡ ‡	57 (41)	23 (45)	16 (39)	18 (38)	0.73 (0.33, 1.64) p=0.449	0.78 (0.34, 1.81) p=0.566	0.76 (0.38, 1.52) p=0.432	0.85 (0.57, 1.28) p=0.446
Myocardial haemorrhage extent (% LV) * ‡‡‡	2.0 ± 3.9	1.6 ± 2.8	2.1 ± 3.8	2.4 ± 4.8	0.72 (-0.87, 2.31) p=0.373	0.44 (-1.28, 2.16) p=0.619	0.60 (-0.81, 2.01) p=0.403	0.36 (-0.43, 1.15) p=0.372
Myocardial hemorrhage presence * ‡ ‡	56 (41)	22 (45)	16 (41)	18 (38)	0.73 (0.33, 1.66) p=0.458	0.85 (0.36, 2.00) p=0.713	0.79 (0.38, 1.60) p=0.506	0.86 (0.57, 1.29) p=0.458
Acute Infarct size (% LV) * ‡‡‡	24.1 ± 12.7	23.3 ± 12.9	26.6 ± 12.5	23.1 ± 12.8	-0.70 (-5.35, 3.94) p=0.767	2.57 (-2.29 7.42) p=0.300	0.80 (-3.27, 4.87) p=0.700	-0.33 (-2.66, 2.00) p=0.781
LV ejection fraction (%) * ‡‡‡	43.9 ± 8.3	44.3 ± 7.6	43.6 ± 7.6	43.7 ± 9.7	-0.32 (-3.48, 2.84) p=0.844	-0.25 (-3.55, 3.05) p=0.882	-0.29 (-3.04, 2.47) p=0.838	-0.16 (-1.74, 1.42) p=0.842
LV end systolic volume (ml), * median (IQR) ‡ ‡‡ ‡	90.5 (77.5, 108.3)	90.4 (80.9, 108.8)	92.9 (83.2, 106.8)	89.9 (66.1, 108.3)	0.94 (0.84, 1.06) p=0.306	1.00 (0.88, 1.12) p=0.946	0.97 (0.87, 1.07) p=0.503	0.97 (0.92, 1.03) p=0.308
LV end diastolic volume (ml), * median (IQR) ‡ ‡‡ ‡	166.0 (143.5, 188.4)	168.7 (151.3, 196.5)	73.5 (147.1, 187.9)	157.9 (131.6, 187.1)	0.94 (0.86, 1.03) p=0.189	0.99 (0.90, 1.09) p=0.850	0.96 (0.89, 1.04) p=0.360	0.97 (0.93, 1.01) p=0.190
**CMR 3 months**								
Infarct size (% LV) * ‡‡‡	17.0 ± 11.5	17.0 ± 11.9	17.7 ± 11.0	16.5 ± 11.7	-0.62 (-5.04, 3.80) p=0.782	0.45 (-4.16, 5.06) p=0.848	-0.13 (-4.00, 3.74) p=0.947	-0.31 (-2.52, 1.89) p=0.780

Myocardial salvage index * ‡‡‡	0.6 ± 0.2	0.6 ± 0.3	0.6 ± 0.2	0.6 ± 0.2	0.02 (-0.08, 0.12) p=0.707	0.02 (-0.08, 0.13) p=0.670	0.02 (-0.07, 0.11) p=0.642	0.01 (-0.04, 0.06) p=0.708
LV ejection fraction * ‡‡‡	49.1 ± 8.4	49.5 ± 8.4	49.0 ± 6.5	48.9 ± 9.8	-0.45 (-3.77, 2.88) p=0.793	-0.32 (-3.79, 3.14) p=0.855	-0.39 (-3.29, 2.51) p=0.792	-0.22 (-1.88, 1.43) p=0.792
LV end systolic volume (ml), * median (IQR) ‡‡‡‡	81.1 (65.7, 102.2)	82.5 (69.0, 99.8)	81.8 (70.1, 92.9)	73.4 (61.6, 109.6)	0.95 (0.83, 1.09) p=0.484	0.97 (0.84, 1.12) p=0.687	0.96 (0.85, 1.08) p=0.513	0.98 (0.91, 1.05) p=0.482
LV end diastolic volume (ml), * median (IQR) ‡‡‡‡	162.8 (141.6, 186.2)	165.6 (149.2, 188.9)	164.6 (147.0, 176.9)	151.6 (132.7, 183.3)	0.93 (0.85, 1.02) p=0.140	0.96 (0.87, 1.06) p=0.403	0.95 (0.87, 1.02) p=0.170	0.97 (0.92, 1.01) p=0.138
Change in LV end diastolic volume at 3 months from 2-7 days (ml), * median (IQR) ‡‡‡‡	-3.5 (-13.5, 10.0)	1.7 (-12.0, 14.6)	-4.7 (-13.1, 6.2)	-4.3 (-16.4, 12.8)	-7.80 (-17.82, 2.21) p=0.127	-9.53 (-19.98, 0.92) p=0.074	-8.60 (-17.34, 0.15) p=0.054	-3.92 (-8.93, 1.10) p=0.126

‡ Data analyzed on square root scale. Treatment effect estimates reported as mean differences in square root of MVO extent between groups, with 95% confidence interval and p-value, from linear regression model adjusted for MI location.

‡‡ Treatment effect estimates reported as odds ratios between groups, with 95% confidence interval and p-value, from a logistic regression model, adjusting for MI location.

‡‡‡ Treatment effect estimates reported as mean differences between groups, with 95% confidence interval and p-value, from linear regression model adjusted for MI location.

‡‡‡‡ Treatment effect estimates reported as relative differences between groups, on a log-transformed scale, with 95% confidence interval and p-value, from linear regression model adjusted for MI location.

*Missing data: Change in LV end diastolic volume at 3 months from 2 – 7 days, 9 subjects (5 placebo, 1 alteplase 10mg, 3 alteplase 20mg). Infarct size, LV ejection fraction, 2 – 7 days post-PCI, 4 subjects (2 placebo, 2 alteplase 20mg group). LV ejection fraction, 3 months post-PCI, 9 subjects (5 placebo, 1 alteplase 10mg, 3 alteplase 20mg group). Infarct size/ myocardial salvage index 3 months post-PCI, 11 subjects (7 placebo, 1 alteplase 10mg, 3 alteplase 20mg group). LV end diastolic/ systolic volume 2 – 7 days post-PCI, 4 subjects (2 placebo, 2 alteplase 20mg). LV end diastolic/ systolic volume 3 months post-PCI, 9 subjects (5 placebo, 1 alteplase 10mg, 3 alteplase 20mg). Microvascular obstruction extent/ presence, infarct size, LV ejection fraction, 4 subjects (2 placebo, 2 alteplase 20mg group). Myocardial hemorrhage extent 13 subjects (6 placebo, 5 alteplase 10mg, 2 alteplase 20mg group). Myocardial hemorrhage extent, 13 subjects (6 placebo, 5 alteplase 10mg, 2 alteplase 20mg). Myocardial hemorrhage presence/ absence, 8 subjects (8 placebo, 2 alteplase 10mg, 2 alteplase 20mg group †Microvascular obstruction was presented as mean ± SD, because the high proportion of patients with a 0 value for microvascular obstruction amount resulted in median values for microvascular obstruction of 0 in each group.

Table S2. Coagulation and hematological variables. Data are mean ± SD, or n (%), unless otherwise stated. Between-group comparisons derived from linear, logistic, or ordinal logistic regression models, adjusted for location of MI (see footnotes).

	Treatment Group				Treatment effect			
	All<br>[n=144]	Placebo<br>[n=53]	Alteplase 10mg<br>[n=41]	Alteplase 20mg<br>[n=50]	20mg vs. placebo<br>Estimate (95% CI)<br>p-value	10mg vs. placebo<br>Estimate (95% CI)<br>p-value	10 or 20mg vs. placebo<br>Estimate (95% CI)<br>p-value	Trend with dos[illegible]<br>Estimate (95% C[illegible]<br>p-value
Hemoglobin (g/L) at 24 hrs * ‡	142.1 ± 12.5	143.5 ± 10.8	141.1 ± 12.1	141.6 ± 14.5	-1.98 (-6.90, 2.94)<br>p=0.431	-2.61 (-7.72, 2.49)<br>p=0.316	-2.28 (-6.55, 2.00)<br>p=0.297	-1.00 (-3.46, 1.4[illegible]<br>p=0.423
Activated clotting time (s) at 2 hrs * ‡‡	288.9 ± 92.0	302.3 ± 103.3	298.3 ± 83.1	266.9 ± 83.5	0.89 (0.80, 1.00)<br>**p=0.046**	1.00 (0.89, 1.13)<br>p=0.940	0.94 (0.85, 1.04)<br>p=0.230	0.94 (0.89, 1.00[illegible]<br>**p=0.048**
Fibrinogen 2 hrs post-PCI * ‡‡‡	3.3 ± 0.9	3.4 ± 1.0	3.3 ± 1.1	3.3 ± 0.6	1.00 (0.90, 1.11)<br>p=0.960	0.98 (0.88, 1.09)<br>p=0.703	0.99 (0.90, 1.08)<br>p=0.809	1.00 (0.95, 1.05[illegible]<br>p=0.954
Plasminogen (U/dL) 2 hrs post-PCI *‡	89.4 ± 14.7	95.3 ± 14.3	90.1 ± 12.4	82.5 ± 14.2	-12.9 (-18.4, -7.30)<br>**p<0.001**	-5.20 (-11.00, 0.60)<br>p=0.076	-9.40 (-14.30, -4.40)<br>**p=0.0002**	-6.40 (-9.20, -3.7[illegible]<br>**p<0.001**
Change in plasminogen (U/dL) 2 hrs post-PCI compared to baseline *‡	-5.3 ± 9.0	-0.1 ± 7.8	-4.7 ± 8.0	-11.3 ± 7.3	-11.30 (-14.40, -8.10)<br>**p<0.001**	-4.60 (-7.80, -1.30)<br>**p=0.006**	-8.20 (-11.10, -5.30)<br>**p<0.001**	-5.60 (-7.20, -4.1[illegible]<br>**p<0.001**
D-dimers 2 hrs post-PCI * ‡‡‡	394.1 ± 543.3	142.4 ± 127.3	536.2 ± 695.8	541.7 ± 580.8	3.49 (2.50, 4.87)<br>**p<0.001**	3.23 (2.28, 4.58)<br>**p<0.001**	3.37 (2.52, 4.50)<br>**p<0.001**	1.88 (1.58, 2.23[illegible]<br>**p<0.001**
tPA 2 hrs post-PCI (ng/ml) * ‡‡‡	23.2 ± 54.4	17.4 ± 35.6	15.4 ± 8.0	36.1 ± 84.1	1.55 (1.22, 1.97)<br>**p=0.0004**	1.16 (0.90, 1.49)<br>p=0.244	1.36 (1.10, 1.68)<br>**p=0.005**	1.24 (1.10, 1.40[illegible]<br>**p=0.0004**

Ratio of tPA 2 hrs post-PCI compared to baseline * ‡‡‡	1.58 ± 1.34	1.16 ± 0.27	1.43 ± 0.59	2.17 ± 2.09	1.58 (1.36, 1.84) **p<0.001**	1.20 (1.03, 1.41) **p=0.022**	1.39 (1.21, 1.60) **p<0.001**	1.26 (1.16, 1.35[illegible] **p<0.001**
Prothrombin fragment F_{1+2} (pmol/L) 2 hrs post-PCI, median (IQR) * ‡‡‡	178.8 (133.1, 244.2)	152.0 (118.6, 211.4)	183.0 (141.9, 291.9)	187.3 (150.5, 244.9)	1.24 (1.00, 1.52) **p=0.048**	1.27 (1.02, 1.58) **p=0.034**	1.25 (1.04, 1.50) **p=0.017**	1.11 (1.00, 1.24[illegible] **p=0.046**

‡ Treatment effect estimates reported as mean differences between groups, with 95% confidence interval and p-value, from linear regression model adjusted for MI location.

‡‡ Treatment effect estimates reported as relative differences between groups, with 95% confidence interval and p-value, from linear regression model adjusted for MI location.

‡‡‡ Data analyzed on logarithmic scale. Treatment effect estimates reported as relative difference between groups, with 95% confidence interval and p-value, from linear regression model adjusted for MI location.

* Missing data: Hemoglobin at 24 hours, 6 subjects (3 placebo, 3 alteplase 20mg group). Activated clotting time, 6 subjects (5 from placebo, 1 from alteplase 10mg group). tPA/ fibrinogen/ d-dimer 2 hours post-PCI, 10 subjects (4 placebo, 2 alteplase 10mg, 4 alteplase 20mg group). tPA ratio 2 hours post-PCI compared to baseline, 15 subjects (6 placebo, 3 alteplase 10mg, 6 alteplase 20mg group). Prothrombin fragment F_{1+2} two hours post-PCI, 15 subjects (6 placebo, 3 alteplase 10mg, 4 alteplase 20mg group). Plasminogen 2 hours post-PCI, 10 subjects (4 placebo, 2 alteplase 10mg. 4 alteplase 20mg group). Change in plasminogen 2 hours post-PCI compared to baseline 15 subjects (6 placebo, 3 alteplase 10mg, 6 alteplase 20mg).

Table S3. IMR, CFR and RRR in sub-groups of TIMI coronary flow grade immediately before study drug delivery. Data are median (IQR). Data analyzed on logarithmic scale. Treatment effect estimates reported as relative differences, derived from linear regression models, adjusted for location of MI.

**TIMI flow grade pre-study drug**	**Placebo**	**Alteplase 10mg**	**Alteplase 20mg**	**Treatment Effect**		**Interaction p-value (treatment as 3-level categorical variable)**	**Treatment Effect**	**Interaction p-value (treatment as 2-level categorical variable)**	**Treatment Effect**	**Interaction p-value (treatment as per 10mg increase in dose)**
	**[n=53]**	**[n=41]**	**[n=50]**	**20mg vs. placebo**	**10mg vs. placebo**		**10 or 20mg vs. placebo**		**Trend with dose**	
**IMR** ≤ 2 [n]	40.5 (29.5, 61.0)<br>[16]	50.5 (26.5, 69.8)<br>[12]	50.5 (25.8, 77.0)<br>[18]	1.05 (0.65, 1.71)<br>p=0.835	1.03 (0.61, 1.77)<br>p=0.903	0.481	1.05 (0.68, 1.62)<br>p=0.834	0.473	1.03 (0.81, 1.31)<br>p=0.828	0.883
3 [n]	28.0 (16.0, 50.5)<br>[35]	19.0 (16.0, 30.0)<br>[29]	30.0 (17.5, 46.5)<br>[31]	1.02 (0.72, 1.45)<br>p=0.896	0.72 (0.51, 1.02)<br>p=0.835		0.86 (0.64, 1.16)<br>p=0.335		1.00 (0.84, 1.20)<br>p=0.959	
**CFR** ≤ 2 [n]	1.6 (1.2, 2.1)<br>[16]	1.4 (1.2, 1.8)<br>[12]	1.3 (1.0, 2.0)<br>[18]	0.84 (0.64, 1.10)<br>p=0.198	0.88 (0.65, 1.18)<br>p=0.396	0.159	0.85 (0.67, 1.09)<br>p=0.201	0.074	0.92 (0.80, 1.05)<br>p=0.198	0.056
3 [n]	1.3 (1.1, 1.8)<br>[35]	1.4 (1.1, 2.1)<br>[29]	1.6 (1.3, 2.0)<br>[31]	1.16 (0.95, 1.41)<br>p=0.139	1.07 (0.88, 1.31)<br>p=0.472		1.12 (0.95, 1.32)<br>p=0.192		1.08 (0.98, 1.18)<br>p=0.136	
**RRR** ≤ 2 [n]	2.0 (1.4, 2.3)<br>[16]	1.9 (1.4, 2.3)<br>[12]	1.5 (1.1, 2.2)<br>[18]	0.82 (0.62, 1.08)<br>p=0.158	0.90 (0.66, 1.23)<br>p=0.502	0.140	0.85 (0.66, 1.09)<br>p=0.203	0.065	0.90 (0.79, 1.04)<br>p=0.155	**0.046**
3 [n]	1.6 (1.3, 2.2)<br>[35]	1.6 (1.4 2.6)<br>[29]	2.0 (1.5, 2.4)<br>[31]	1.16 (0.95, 1.41)<br>p=0.154	1.10 (0.90, 1.35)<br>p=0.341		1.13 (0.95, 1.34)<br>p=0.160		1.08 (0.97, 1.19)<br>p=0.148	

Table S4. IMR, CFR and RRR in sub-groups of TIMI thrombus grade immediately before study drug delivery. Data are median (IQR). Data analyzed on logarithmic scale. Treatment effect estimates reported as relative differences, derived from linear regression models, adjusted for location of MI.

Thrombus grade pre-study drug	Placebo [n=53]	Alteplase 10mg [n=41]	Alteplase 20mg [n=50]	Treatment Effect 20mg vs. placebo	Treatment Effect 10mg vs. placebo	Interaction p-value (treatment as 3-level categorical variable)	Treatment Effect 10 or 20mg vs. placebo	Interaction p-value (treatment as 2-level categorical variable)	Treatment Effect Trend with dose	Interaction p-value (treatment as per 10mg increase in dose)
**IMR** ≤ 2 [n]	21.0 (15.0, 49.0) [15]	39.0 (17/0, 44.0) [9]	37.0 (20.0, 45.0) [9]	1.33 (0.72, 2.44) p=0.363	1.24 (0.67, 2.29) p=0.498	0.260	1.28 (0.77, 2.13) p=0.348	0.158	1.16 (0.85, 1.57) p=0.353	0.158
≥ 3 [n]	39.5 (21.8, 57.2) [36]	20.0 (16.0, 33.2) [32]	38.5 (19.8, 58.2) [40]	0.97 (0.70, 1.36) p=0.865	0.69 (0.48, 0.98) p=0.036		0.83 (0.62, 1.12) p=0.231		0.99 (0.84, 1.17) p=0.231	
**CFR** ≤ 2 [n]	1.3 (1.1, 2.1) [15]	1.1 (1.1, 1.6) [9]	1.8 (1.5, 2.0) [9]	1.06 (0.76, 1.48) p=0.737	0.86 (0.62, 1.21) p=0.393	0.506	0.96 (0.73, 1.26) p=0.759	0.616	1.02 (0.86, 1.20) p=0.843)	0.976
≥ 3 [n]	1.3 (1.1, 1.8) [36]	1.4 (1.3, 2.1) [32]	1.4 (1.2, 2.0) [40]	1.03 (0.86, 1.23) p=0.753	1.05 (0.87, 1.28) p=0.595		1.04 (0.89, 1.22) p=0.637		1.01 (0.93, 1.11) p=0.765	
**RRR** ≤ 2 [n]	1.6 (1.3, 2.5) [15]	1.4 (1.2, 2.0) [9]	2.0 (1.7, 2.0) [9]	1.05 (0.75, 1.48) p=0.767	0.87 (0.61, 1.23) p=0.424	0.440	0.96 (0.72, 1.27) p=0.768	0.595	1.01(0.86, 1.20) p=0.865	0.948
≥ 3 [n]	1.6 (1.4, 2.2) [36]	1.8 (1.5, 2.7) [32]	1.7 (1.3, 2.4) [40]	1.02 (0.85 1.23) p=0.840	1.09 (0.89, 1.33) p=0.412		1.05 (0.89, 1.24) p=0.580		1.01 (0.92, 1.11) P=0.863	

Supplemental References:

1. Group TS. The Thrombolysis in Myocardial Infarction (TIMI) trial. Phase I findings. The New England journal of medicine 1985;312:932-6.
2. Gibson CM, Murphy SA, Rizzo MJ, Ryan KA, Marble SJ, McCabe CH, Cannon CP, Van de Werf F, Braunwald E. Relationship between TIMI frame count and clinical outcomes after thrombolytic administration. Thrombolysis In Myocardial Infarction (TIMI) Study Group. Circulation 1999;99:1945-50.
3. Gibson CM, de Lemos JA, Murphy SA, Marble SJ, McCabe CH, Cannon CP, Antman EM, Braunwald E; TIMI Study Group. Combination therapy with abciximab reduces angiographically evident thrombus in acute myocardial infarction: a TIMI 14 substudy. Circulation 2001;103:2550-4.
4. Sorensson P, Heiberg E, Saleh N, Bouvier F, Caidahl K, Tornvall P, Rydén L, Pernow J, Arheden H. Assessment of myocardium at risk with contrast enhanced steady-state free precession cine cardiovascular magnetic resonance compared to single-photon emission computed tomography. Journal of cardiovascular magnetic resonance : official journal of the Society for Cardiovascular Magnetic Resonance 2010;12:25.
5. Anderson LJ, Holden S, Davis B, Prescott E, Charrier CC, Bunce NH, Firmin DN, Wonke B, Porter J, Walker JM, Pennell DJ. Cardiovascular T2-star (T2*) magnetic resonance for the early diagnosis of myocardial iron overload. European heart journal 2001;22:2171-9.
6. Carrick D, Haig C, Ahmed N, Ahmed N, McEntegart M, Petrie MC, Eteiba H, Hood S, Watkins S, Lindsay MM, Davie A, Mahrous A, Mordi I, Rauhalammi S, Sattar N, Welsh P, Radjenovic A, Ford I, Oldroyd KG, Berry C. Myocardial Hemorrhage After Acute Reperfused ST-Segment-Elevation Myocardial Infarction: Relation to Microvascular Obstruction and Prognostic Significance. Circulation Cardiovascular imaging 2016;9:e004148.
